# Advanced penile cancer – a very sad reality in developing countries

**DOI:** 10.1590/S1677-5538.IBJU.2021.06.01

**Published:** 2021-10-01

**Affiliations:** 1 Universidade do Estado do Rio de Janeiro Unidade de Pesquisa Urogenital Rio de JaneiroRJ Brasil Unidade de Pesquisa Urogenital - Universidade do Estado do Rio de Janeiros - Uerj, Rio de Janeiro, RJ, Brasil; 2 Hospital Federal da Lagoa Serviço de Urologia Rio de JaneiroRJ Brasil Serviço de Urologia, Hospital Federal da Lagoa, Rio de Janeiro, RJ, Brasil

The November-December 2021 number of *Int Braz J Urol*, the 14th under my supervision, presents original contributions with a lot of interesting papers in different fields: Overactive Bladder, Penile Cancer, Prostate Cancer, Male Urinary Incontinence, Kidney Stones, LUTS, Ureteropelvic Junction, Testicular Torsion, Obstruction Renal Cell Carcinoma, Bladder Cancer and Infertility. The papers came from many different countries such as Brazil, USA, Portugal, China, Singapore, Germany, France, Colombia and Italy and as usual the editor´s comment highlights some of them.

In the present issue we will comment three important papers about penile cancer. Dr. Azevedo and colleagues from Brazil perfomed in page 1108 (1) a nice narrative review about the use of flaps in inguinal lymphadenectomy in metastatic penile câncer and concluded that several fasciocutaneous and myocutaneous flaps of the abdomen and thigh can be used for the reconstruction of the inguinal region and that the reconstruction of defects in the inguinal region with the aid of flaps allows for faster postoperative recovery and reduces the risk of complications. Dr. Koifman and colleagues from Brazil performed in page 1162 (2) an interesting original study about the role of primary inguinal surgical debulking for locally advanced penile cancer followed by reconstruction with myocutaneous flap. This paper is the cover in this number. The authors concluded that primary radical inguinal surgical debulking alone for advanced loco-regional penile cancer is unlikely to promote long term survival, although it can lead to temporary local control of the disease. Despite the feasibility of the procedure, it is related to high incidence of complications. Surgical treatment with adjuvant chemotherapy is associated with improved overall survival and the 3rd paper about penile cancer in this number, was performed by Dr. Garcia and colleagues from Colombia in page 1259 (3). In this interesting comment the authors concluded that the population living in rural areas might go through different environmental and behavioral factors delaying diagnosis and treatment, such as accessing the health system, knowledge about the natural history, risky sexual intercourse, and higher prevalence of HPV infection. Accordingly, penile cancer needs an early diagnosis and treatment without delays. The editor in chief would like to highlight the following works too:

Dr. Kreydin and colleagues from USA, Brazil and Portugal performed in page 1091 (4) a very important narrative review about the current pharmacotherapy of overactive bladder and comment that various antimuscarinic agents and the beta-3 agonists mirabegron and vibegron are currently available for the treatment of overactive bladder (OAB). The authors concluded that lower urinary tract sensation and contractility are mediated by a multitude of mechanisms and receptors. Some of these are being investigated as potential targets for novel oral therapies for OAB; Altering afferent bladder signaling may be a novel approach to OAB therapy; Agonists and inhibitors of pain and mechanotransduction receptors such as TRPV and cannabinoid receptors are currently in preclinical and clinical studies and have shown some promise in certain patient populations.

Dr. Guo and colleagues from China and Singapore performed in page 1120 (5) a nice sistematic review about the periodontal disease and the risk of prostate cancer and concluded that periodontal disease was associated with the increased risk of prostate cancer, whereas no significant association was observed in patients treated with periodontal therapy. Hence, the awareness and importance for maintaining oral health should be improved, and the underlying mechanisms linking periodontal disease and prostate cancer should be fully explored in future research.

Dr. Inouye and colleagues from USA performed in page 1131 (6) a nice review about the male sling for stress urinary incontinence and concludede that male urethral slings are a great surgical option for the patient with mild stress urinary incontinence without a history of radiation therapy and one of the most important components of success with this type of procedure is appropriate patient selection.

Dr. Danilovic and colleagues from Brazil performed in page 1136 (7) a interesting study about the effect of a low-calorie diet on 24-hour urinary parameters of obese adults with idiopathic calcium oxalate kidney stones and concluded that short-term modest weight loss induced by twelve weeks of low-calorie diet is not associated with a decrease of 24-hour urinary lithogenic parameters in idiopathic calcium oxalate stone formers. Calcium oxalate urinary stone formation is probably multifactorial and driven by other factors than weight.

Dr. Yildiz and colleagues from Turkey performed in page 1150 (8) a prospective randomized controlled trial about the efficacy of intravaginal electrical stimulation added to bladder training in women with idiopathic overactive bladder and concluded that bladder training and intravaginal electrical stimulation were more effective than bladder training alone on both incontinence-related QoL and clinical parameters in women with idiopathic OAB.

Dr. Gondim and colleagues from Brazil performed in page 1178 (9) a interesting study about the evaluation of autonomic function in children and adolescents with overactive bladder and concluded that t he capacity for coordinated sympathetic and parasympathetic activity during the micturition process was found to be better in the control group, with a predominance of sympathetic activity during the bladder-filling phase and better heart rate variability.

Dr. Rychik and colleagues from USA, performed in page 1189 (10) a nice study about the relationship between maximum voided volume obtained by bladder diary compared to contemporaneous uroflowmetry in men and women and concluded that there is a difference between the two measurement tools, and that the 24hour bladder diary-maximum voided volume (BD-MVV) was greater than maximum voided volume determined at the time of uroflowmetry (Q-MVV). For a more reliable assessment of MVV, this study suggests that both Q-MVV and BD-MVV should be assessed and that the larger of the two values is a more reliable assessment of MVV.

We hope that readers will enjoy the present number of the International Brazilian Journal of Urology.
